# Realizing Function by Carbon–Rich Molecular Architectures for Future Technologies

**DOI:** 10.1002/advs.202203029

**Published:** 2022-07-06

**Authors:** Andreas Herrmann, Tanja Weil

This special issue aims to celebrate the 75th birthday of Professor Klaus Müllen and highlights his achievements and impact on a wide range of areas involving functional carbon‐rich materials. We would like to honor a remarkable chemist, a visionary researcher and an inspiring teacher for several generations of chemists and materials scientists worldwide, who has stimulated and shaped this research field over several decades.


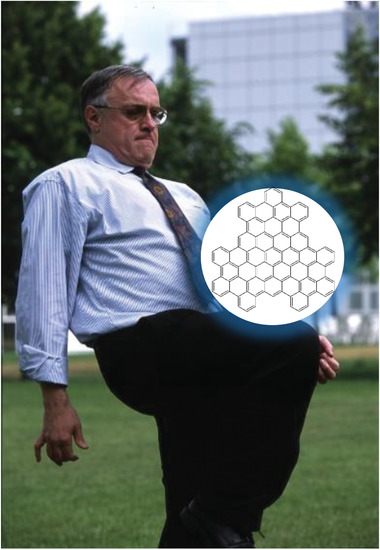


Klaus Müllen began his scientific career at the University of Cologne with Prof. Emanuel Vogel. In 1972, he received his doctorate from the University of Basel with Prof. Fabian Gerson and continued his postdoctoral work at the ETH Zurich in the laboratory of Prof. Jean François Michel Oth, where he habilitated in 1977. At this early stage in his career, he was largely engaged in physical chemistry, using electron magnetic resonance spectroscopy and dynamic nuclear magnetic resonance spectroscopy to characterize molecular structures. He then received professorships at the Universities of Cologne and Mainz before becoming Director at the Max Planck Institute for Polymer Research in Mainz in 1989. During this time, he moved into the area of aromatic and carbon‐rich molecules, laying the foundations for the generation of several novel classes of molecular architectures. After his retirement in early 2016, he continued his research with an emeritus group funded by the Max Planck Society as well as a Fellowship of the Johannes Gutenberg Research College at the University of Mainz.

During his career, Prof. Müllen realized many ground‐breaking innovations in materials chemistry, especially in the areas of conjugated polymers, organic dyes, polymer synthesis and dendrimers. His work has been published in more than 2100 journal articles and 70 patents. It is therefore impossible to give an exhaustive overview of his life's work here. As such, we would like to highlight just one of the areas where we believe Prof. Müllen has had a tremendous impact on the scientific community. With the design, synthesis, characterization and the later resulting applications, he entered completely new scientific grounds and this topic of nanographenes is also the subject of several articles in this special issue. His early efforts in the 1990s to evolve conjugated polymers into nanographenes were very visionary. At that time, he dedicated his work to “ladder‐type” conjugated polymers in which adjacent benzene rings are forced into a fully planar conformation, such as found in single‐layer graphene. Next, taking inspiration from organic pigments and dyes, he developed more extended planar *π*‐conjugated systems that represent the homologue series of poly‐peri‐naphthalenes. The well‐known high‐performance pigment perylenediimide was successively expanded to quarter‐, penta‐ and hexarylene with four, five or six planar fused naphthalene units. This class of dyes is highlighted in this collection of articles, and both structures, i.e., ladder‐type conjugated polymers and oligorylenes, can be considered as the first steps towards graphene nanoribbons.

Beyond one‐dimensional *π*‐systems, Klaus Müllen pioneered two‐dimensional (2D) graphene structures. The first example represents hexabenzocoronene, which can be viewed as a well‐defined graphene island composed of seven benzene rings forming a flat disk. An important feature of this material is that it is solution processable due to peripheral alkyl chain modifications. Not only did his group successfully master the synthesis of these sophisticated nanostructures, Klaus Müllen also recognized the application potential of these materials for electronic applications. Therefore, several articles in this special issue deal with the characterization and application of these molecules in electronic devices. In 1995, it was demonstrated that nanographenes could be divided into conductive and non‐conductive areas, from which currents could be measured with submolecular spatial resolution. These discoveries were made long before individual free‐standing graphene sheets were visualized and used for physical experiments.

After that, the Müllen laboratory prepared a wide variety of nanographenes varying shapes and functionalities, which also led to the discovery of graphene nanoribbons (GNRs). GNRs are currently receiving tremendous attention for opening the band gap of graphenes in a controlled manner, making them suitable as another class of polymeric semiconductors in devices. Equally important are nonplanar graphenes and graphenes in which defined carbon atoms are replaced by heteroatoms. In analogy to inorganic semiconductors, one can speak here of molecular doping of 2D polymers. Nitrogen‐doped graphene molecules have been successfully used for oxygen reduction in fuel cells, and graphene‐metal oxide hybrid materials have been used as anode materials for lithium‐ion batteries, both with exceptional performance. It is therefore not surprising that many of the molecules described above have been workhorses in a large number of laboratories around the world.

A central topic of Klaus Müllen's research is the control of function through molecular design, enabled by novel molecules, macromolecules, and 2D and 3D nanomaterials that have been applied in various fields. Without question, these *π*‐ and carbon‐rich structures have been an inspiration to many scientists, as evidenced by more than 180000 citations, making him the most‐cited German chemist. His work has received numerous prizes such as the Karl Ziegler Prize from the Society of German Chemists, the Cothenius Medal from the National Academy of Sciences Leopoldina, the Spiers Memorial Award from the Royal Society of Chemistry and the Leonardo da Vinci Award from the European Academy of Sciences, just to name a few recent ones. His impact on the scientific community is equally evident in many honorary doctorates and editorial board memberships, including in the Wiley Advanced Materials family.

During his career, Klaus Müllen supervised more than 300 doctoral students and 180 post‐docs, of which more than 70 hold academic positions. This impressively shows his inspiring character, his ability to motivate and stimulate unconventional thinking as well as his great support of next generation scientists. He has always been an extremely stimulating and creative mentor, fueling an environment that encourages young collaborators to engage in interdisciplinary research in diverse teams. At the center of all work was a scientific discussion, in which he listened and spiced the interaction with competence, creativity, intelligence and great scientific intuition. This paradigm and creative process is carried further by its alumni to drive future innovation beyond the field of chemistry.

This special issue focuses on the synthesis of carbon‐rich materials and highlights their unique properties, which derive from their molecular architecture and enable diverse functionalities in different fields such as optics, electronics, but also in biology and medicine. For this issue, we have invited close collaborators of Klaus Müllen and former alumni who have carried Klaus Müllen's spirit further. We are very grateful for their contributions.

We greatly appreciate the help of Dr. Kirsten Severing, Dr. Anne Pfisterer, Anke Osterland and the Advanced Science editorial team for putting this special issue together, and we would like to thank once again all the scientists who contributed their scientific findings and opinions to show their appreciation for Klaus Müllen. Finally, we wish Klaus a happy birthday!

